# Contrasting effects of exogenous phosphorus application on N_2_O emissions from two tropical forest soils with contrasting phosphorus availability

**DOI:** 10.1186/s40064-016-2587-5

**Published:** 2016-08-02

**Authors:** Taiki Mori, Daiki Yokoyama, Kanehiro Kitayama

**Affiliations:** 1 Forest Ecology Laboratory, Graduate School of Agriculture, Kyoto University, Kitashirakawa Oiwake-cho, Sakyo-ku, Kyoto, 606-8502 Japan; 2 Key Laboratory of Vegetation Restoration and Management of Degraded Ecosystems, South China Botanical Garden, Chinese Academy of Sciences, Guangzhou, 510650 Guangdong China

**Keywords:** Nitrous oxide, Phosphorus limitation, Tropics, Nitrification, Denitrification

## Abstract

An incubation study was conducted to test the effects of phosphorus (P) addition on nitrous oxide (N_2_O) emissions from the soils taken from two tropical rain forests established on different parent materials [meta-sedimentary (MS) and ultrabasic (UB) rock] on Mt. Kinabalu, Borneo. Earlier studies suggest that the forest on UB soils is more strongly limited by P than that on MS soils is. In MS soils, P addition significantly reduced N_2_O emissions. Since neither ammonium (NH_4_^+^) nor nitrate (NO_3_^−^) contents were reduced by P addition, we assumed that the decrease in N_2_O emissions were not due to the previously-reported mechanism: P addition stimulated microbial nitrogen (N) immobilization and collateral inorganic N consumption, reducing resources for producing N_2_O. Since P addition enhanced the ratios of microbial biomass to CO_2_ and N_2_O emissions (indicators of nitrifying and/or denitrifying respiratory efficiency), it was suggested that the N required for the respiration of nitrifying and/or denitrifying bacteria was reduced, leading to reduced N_2_O emissions. On the other hand, P addition had no effects on N_2_O emissions in UB soils. The respiratory efficiency did not change significantly by P addition, possibly because the microbial community in the highly-P-depleted UB soils shifted by P addition, with which the enhancement of respiration efficiency did not co-vary. We concluded that (1) P addition may control N_2_O emissions through increasing respiratory efficiency, and (2) the effects may be different depending on the differences in P availability.

## Background

Nitrous oxide (N_2_O) is the third most important global warming gas (IPCC 2007) and also the most important ozone-depleting gas (Ravishankara et al. 2009). Soils are one of the major sources for N_2_O, which is a by-product or an intermediate of microbial nitrification and denitrification, respectively (Firestone and Davidson 1989; Wrage et al. 2001; Ishizuka et al. 2002; Keller et al. 2005). Various factors are suggested to control N_2_O emission including direct factors such as the availability of soil inorganic nitrogen (N) (Firestone and Davidson 1989; Davidson and Verchot 2000; Arai et al. 2008; Liu and Greaver 2009) and organic carbon (C) (Nobre et al. 2001), soil temperature (Cavelier et al. 2000; Dobbie and Smith 2001; Schindlbacher and Zechmeister-Boltenstern 2004), moisture (Klemedtsson et al. 1988; Firestone and Davidson 1989; Davidson and Verchot 2000; Erickson et al. 2001; Konda et al. 2010), bulk density (Sitaula et al. 2000), and pH (Kesik et al. 2006; Baggs et al. 2010), and indirect factors such as land use (Ishizuka et al. 2002), land use history (Van Lent et al. 2015), vegetation (Erickson et al. 2001; Konda et al. 2008), and soil parent material (Hall et al. 2004).

In tropical forest ecosystems, which account for 14–23 % of the current N_2_O budget (IPCC 2007), phosphorus (P) availability may be another important factor controlling N_2_O emissions. Phosphorus is generally believed to be the main limiting factor in tropical forest ecosystems on Ultisoils and Oxisols due to the low P supply from highly weathered soil and relatively high N input (Walker and Syers 1976; Elser et al. 2007; Vitousek et al. 2010). Microbial activity including nitrification or denitrification is also suggested to be limited by P availability (Minami and Fukushi 1983; Kitayama et al. 1997, 1998; Cleveland et al. 2002; Ilstedt et al. 2003; Kitayama et al. 2004; Ilstedt and Singh 2005; Cleveland and Townsend 2006; Mori et al. 2010b, 2013a; He and Dijkstra 2015).

Recently several studies reported that P application reduced N_2_O emissions. They suggested that added-P stimulated plant N uptake and reduced N resources for N_2_O production (Mori et al. 2013b; Baral et al. 2014; Zhang et al. 2014; Chen et al. 2015). This idea was experimentally confirmed by Mori et al., demonstrating that P addition reduced N_2_O emissions from *Acacia mangium* plantation sites with roots, while conversely stimulated the emissions from root-excluded sites (Mori et al. 2014).

Contrasting with the observations in the field with vegetation, results regarding how P controls the microbial activity (without the interference of vegetation) and accompanying N_2_O emissions are not consistent. Hall and Matson (1999) observed that N addition to P-limited forest soils generated 10–100 times higher N_2_O fluxes than to N-limited forest soils. They also demonstrated that the ^15^N-labeled inorganic N added to the N-limited soils readily became a microbial form, while that added to the P-limited soils largely remained as inorganic form. From these results, they suggested that P shortage in tropical soils restricts microbial N immobilization, which supplies more N sources for nitrification and/or denitrification, stimulating N_2_O emissions (Hall and Matson 1999). Sundareshwar et al. (2003) demonstrated that N_2_O emissions from sediments from a coastal salt marsh in South Carolina decreased by P addition because of an increase in N immobilization and a subsequent decrease in denitrification (Sundareshwar et al. 2003). On the other hand, Mori et al. reported that P addition stimulated N_2_O emissions both from nitrification and denitrification (Mori et al. 2010a, 2013c). They attributed the results to the following mechanisms: (1) P addition directly stimulated nitrifying and/or denitrifying activities; and (2) P addition stimulated heterotrophic CO_2_ consumption and promoted a more reductive condition, which produces more N_2_O emissions.

Thus, so far, it is not clear how P controls soil microbial activities and accompanying N_2_O emissions in P-limited tropical forest soils. Especially, the reason why N_2_O emissions respond differently to P addition (or P shortage) among studies is unknown. In the present paper we hypothesized that P addition affects N_2_O emissions differently depending on the strength of P shortage. Long term ecological study sites in Mt. Kinabalu (Kitayama and Aiba 2002) is an ideal sites for testing this hypothesis, because the sites consist of two types of study sites on two different soils with different P availability (Kitayama et al. 2004; Kitayama 2013). We conducted an incubation experiment using soils taken from two primary tropical rain forests established on different parent materials [meta-sedimentary (MS) and ultrabasic (UB) rock] on a lower eastern slope of Mt. Kinabalu, Sabah, Malaysia. Earlier studies suggest that the forest on UB soils is more strongly limited by P than that on MS soils is (Kitayama and Aiba 2002).

## Methods

### Field location and soil sampling

The study field is located on the lower eastern slope of Mt. Kinabalu (4095 m, 6°05′N, 160°33′E) within Kinabalu Park, Sabah, Malaysia. We selected a pair of lowland dipterocarp forests established at the same altitude (700 m) with the same rock age (Tertiary) but with contrasting parent materials of MS and UB rocks (Table 1). Both sites are intact primary rain forests with no prior land use history and have similar basal areas and stem densities (Table 1). The climate is aseasonal in monthly temperature with a mean annual temperature of approximately 23.8 °C and precipitation ranging from 2300 to 2500 mm year^−1^ (Aiba and Kitayama 1999). The studied site is a subset of the long-term ecological study described in Kitayama and Aiba (2002). Selected site characteristics are shown in Table 1.

**Table 1:** Site characteristics of sedimentary and ultrabasic sites on Mount Kinabalu, Sabah, Malaysia

	Meta-sedimentary (MS)	Ultrabasic (UB)
Slope (°)^a^	19	11
Basal Area (m^2^ ha^−1^)^a^	36.2	40.7
Stem density (m^2^ ha^−1^)^a^	1064	1175
Above ground biomass (kg m^−2^)^a^	49.1	55.2
ANPP (g m^−2^ year^−1^)^a^	1913	1715
Clay content (%)^b^	37.8	45.6
Soil organic C (%)^a^	2.9	2.4
Soil total N (%)^a^	0.21	0.21
Soil C:N^a^	13.8	11.4
Soil pH in H_2_O^a^	4.1	4.5
Field bulk density^b^	0.89	0.83
Soil P pools per 30 cm (g m^−2^)^c^		
Ca-Pi	1.71	2.07
CO_3_-Pi	0.07	0.22
OH-Pi	8.40	7.50
Occl-Pi	22.19	1.60
CO_3_-Po	2.23	4.71
OH-Po	12.16	13.06
HCl-Po	14.61	0.03
Total Po	28.99	17.79
Total P	61.36	29.19
Bray-1 P (μg P g soil^−2^)^d^	2.18 (0.21)	0.94 (0.06)

^a^From Aiba and Kitayama (1999) and Kitayama and Aiba (2002)

^b^From Wagai et al. (2008) using 0–10 cm surface mineral horizon

^c^From Kitayama et al. (2000) and Kitayama et al. (2004)

^d^The present study. The values indicate the average of 5 replication ± standard error

At each site, we laid five transects (40 m) and took 20 soil cores (0–15 cm) at 2 m intervals from each transect using a stainless soil corer (3.4 cm diameter and 60 cm long). The cored soils were immediately taken back to the laboratory and kept under 4 °C after composited across the 20 soil cores by each transect (yielding a total of five composites) and put through a 2 mm sieve. After sieving, soil bulk density became lower (0.51 and 0.46 in UB soil and MS soil, respectively) than field condition (see Table 1), which may have influenced the microbial activities and gas emissions to some extent. Bray-1 P content in each composited sample was determined (data shown in Table 1) by extracting P after shaking 1 g air-dry soil and 7 ml Bray-1 solution for 1 min (Kuo 1996).

### Incubation

Twenty g fresh soil was placed in a 223 ml wide mouth jar for a gas emission analysis, 5 g in a 50 ml plastic bottle for analyzing inorganic N, dissolved organic C (DOC) and dissolved N (DN), and 5 g in a 50 ml glass bottle for a soil microbial biomass analysis. We prepared two subsamples for each analysis, one for P addition and the other for control. We added P as KH_2_PO_4_ solution (100 μg P g soil^−1^, dissolved in distilled water) to each soil so that soil water condition became equivalent to 80 % water holding capacity (WHC). Controls were prepared without P addition in the same manner. The samples were incubated at 25 °C in the dark for 48 h. In the present study, our purpose was not measuring the precise fluxes of the gases, but comparing gas emissions between P-added soil and non-added control, not missing the emission peaks. Thus we chose to measure gas emissions by closing lids for 48 h. Although gas concentration may have not increased linearly and the differences among treatment may have been underestimated, we considered it more important not to miss the emission peaks. Previous incubation studies showed that N_2_O emissions declined to low level at 1–3 days (Mori et al. 2010a, 2013c). Wide mouth jars were closed with butyl rubber stoppers equipped with sampling ports, and gas samples were taken 0 and 48 h after the closure of the stoppers. N_2_O, NO and CO_2_ emissions were measured by calculating the changes of the gas concentrations during the incubation period. The N_2_O concentration in the gas sample was analyzed using a gas chromatograph (GC-14B, SHIMADZU, Kyoto, Japan) equipped with an electron capture detector. The column, injector, and detector temperatures were kept at 60, 80 and 330 °C, respectively. Argon containing 5 % CH_4_ was used as a carrier gas. The NO concentration was analyzed with a NO–NO_2_–NO_x_ Analyzer (Model 42i, Nippon Thermo Co. Ltd., Kyoto, Japan). The CO_2_ concentration was analyzed with a gas chromatograph (GC-14B, SHIMADZU, Kyoto, Japan) equipped with a thermal conductivity detector, using He as a carrier gas. The column, injector and detector temperatures were kept at 60, 60 and 100 °C, respectively.

Inorganic N (NH_4_^+^ and NO_3_^−^), DOC and DN were extracted at the end of the incubation by shaking 5 g soil with 50 ml 0.5 M K_2_SO_4_ extractant for 30 min. The supernatants were filtered and refrigerated until the analysis. Ammonium was determined by indophenol blue absorptiometry and NO_3_^−^ by naphthyl ethylenediamine method using a flow injection analyzer (AQLA-700-NO, AQUA LAB, Japan). DOC and DN were analyzed by a total organic carbon analyzer with a total organic nitrogen measurement unit (TOC-V_E_/TNM-1, SHIMADZU, Kyoto, Japan). Soil microbial biomass C (MBC) and N (MBN) were determined by a chloroform fumigation extraction method (Vance and Jenkinson 1987). Five g fresh soils were exposed to CHCl_3_ vapor for 24 h in a vacuum desiccator at 25 °C after 48-h incubation. After residual CHCl_3_ was removed, fumigated soils were shaken with 50 ml of 0.5 M K_2_SO_4_ extractant for 30 min and soluble C and N were extracted. Equivalent portions of unfumigated soils were also extracted. Soluble C and N were analyzed on a total organic carbon analyzer with a total organic nitrogen measurement unit (TOC-V_E_/TNM-1, SHIMADZU, Kyoto, Japan). Soil microbial biomass element contents were calculated from the differences between the fumigated and unfumigated samples using a conversion factor of 0.45 (Jenkinson et al. 2004). Since measuring the real C use efficiency or respiratory efficiency was technically difficult (Sinsabaugh et al. 2013), we used the ratio of MBC to CO_2_ or N_2_O (an inverse of respiratory quotient) as indicators of microbial respiratory efficiency. A number of studies have used respiratory quotient as an indicator of microbial C use efficiency (Giller et al. 1998; Priess and Fölster 2001; Schipper and Lee 2004).

### Statistical analysis

Statistical analyses were performed by Excel 2010 with Statcel 3 (OMS Ltd.) (ex. Ohyagi-Hara et al. 2013; Shigenobu et al. 2014; Mori et al. 2015) or Excel 2013 with statistical add-in software (Social Survey Research Information Co., Ltd.) (ex. Mori et al. 2016). The level of significance was examined by a paired *t* test after confirming the normality of data. Some data sets not normally distributing were log-transformed prior to statistical analysis. For some data sets not following the normal or log-normal distribution, we adopted non-parametric Mann–Whitney’s *U* test. Correlation coefficient was obtained sing simple regression analysis.

## Results

**Fig. 1:**
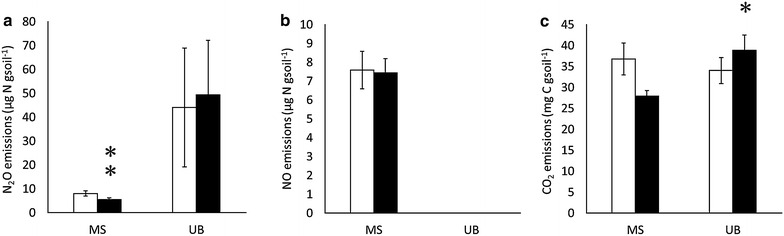
Effects of P addition on cumulative emission of **a** N_2_O, **b** NO, and **c** CO_2_ during 48-h incubation. *P < 0.05; **P < 0.01. *SE* standard error, *MS* meta-sediment rock soil, *UB* ultrabasic rock soil

**Fig. 2:**
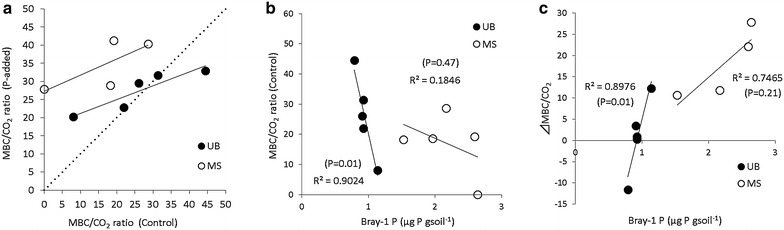
**a** Effects of P addition on the ratio of soil microbial biomass C (MBC) to CO_2_ emissions. **b** Relationship between soil Bray-1 P content and the MBC/CO_2_ ratio in the control soils. **c** Relationship between soil Bray-1 P contents and the differences of the MBC/CO_2_ ratios between control and P added soils (ΔMBC/CO_2_). *MS* meta-sediment rock soil, *UB* ultrabasic rock soil. Because of the experimental failure about CO_2_ measurement analysis in P-added soil, we report only 4 of 5 replications in MS in **a**, **c**

In MS soils, P addition significantly reduced N_2_O emissions (Fig. 1), while it did not change NO or CO_2_ emissions. On the other hand, P addition stimulated CO_2_ emissions in UB soils, but had no significant effects on N_2_O and NO emissions (Fig. 1). N_2_O emissions from UB soils showed higher values than MS soils did, with high variability. We could not observe NO emissions (under detectable level) in UB soils. A higher bulk density (0.51 and 0.46 in UB soil and MS soil, respectively) and a higher clay content (Table 1) in UB soils than MS soils probably provided a more reductive condition in the UB soils, causing a higher N_2_O/NO ratio (Fig. 1). P addition did not change NH_4_^+^, NO_3_^−^, DOC or DN contents at the end of the incubation period (Table 2). Soil microbial biomass (MBC and MBN) tended to increase by P addition in MS soils (P = 0.09 and 0.07 in MBC and MBN, respectively), but not in UB soils (Table 3). P addition increased respiratory efficiency (i.e. the ratio of MBC to CO_2_ or N_2_O) significantly in MS soils, but not in UB soils (Table 3). The inconsistence was due to the differences in the relationship between MBC/CO_2_ with and without P addition (Fig. 2a). In MS soils, MBC/CO_2_ ratio was consistently higher in P-added soils than controls in MS. On the other hand, in UB soils, P addition stimulated MBC/CO_2_ ratio when the intact soil (control) was low in MBC/CO_2_ ratio (lower than 30), and in contrast reduced MBC/CO_2_ ratio when the intact soil (control) was high in MBC/CO_2_ ratio (greater than 30). In the UB soils, higher initial respiratory efficiency (MBC/CO_2_ ratio) was associated with lower soil P availability (Bray-1 P content) with significant correlations (P = 0.01), but the trend was not significant in MS soils (Fig. 2b). The differences of the MBC/CO_2_ ratio between control and P added soils (ΔMBC/CO_2_) were correlated well with Bray-1 P contents, especially in UB soils, with larger ΔMBC/CO_2_ in soils with higher P availability (Fig. 2c).

**Table 2:** Soil C and N properties at the end of the incubation

Soil	Treatment	NH_4_ (μg N g soil^−1^)		NO_3_ (μg N g soil^−1^)		DOC (μg C g soil^−1^)		DN (μg N g soil^−1^)	
		Avr.	SE	Avr.	SE	Avr.	SE	Avr.	SE
Meta-sedimentary (MS)	Control	60.4	10.0	107.4	1.9	772.8	47.5	201.9	15.9
	P-added	84.6	19.6	106.6	2.6	751.3	27.1	196.0	10.4
Ultrabasic (UB)	Control	94.8	8.4	61.4	7.4	419.0	8.4	181.9	10.1
	P-added	111.7	17.4	61.9	7.2	429.3	13.1	180.2	8.3

There were no significant differences between control and P-added soils

*DOC* dissolved organic C, *DN* dissolved N, *SE* standard error

**Table 3:** Soil microbial biomas C, N, and their ratio to CO_2_ and N_2_O emissions

Soil	Treatment	MBC (μg C g soil^−1^)		MBN (μg N g soil^−1^)		MBC/CO_2_ emissions		MBN/N_2_O emissions	
		Avr.	SE	Avr.	SE	Avr.	SE	Avr.	SE
Meta-sedimentary (MS)	Control	599.1	162.7	92.8	31.9	16.9	4.7	75.0	22.5
	P-added	919.3	73.3	163.9	13.2	34.6*	3.6	174.5*	19.2
Ultrabasic (UB)	Control	825.5	130.6	121.7	31.5	26.4	6.0	206.2	123.0
	P-added	1030.2	32.8	201.8	16.3	27.4	2.5	389.8	296.3

*MBC* soil microbial biomass C, *MBN* soil microbial biomass N, *SE* standard error

* P < 0.05

## Discussion

In MS soils, P addition significantly reduced N_2_O emissions (Fig. 1), which is in contradiction to the accounts by Mori et al. (2010a, 2013c). They reported that P addition stimulated N_2_O emissions both from nitrification and denitrification, possibly because of the following two mechanisms: (1) P addition directly activated nitrifying and/or denitrifying bacteria; (2) P addition stimulated O_2_ consumption by heterotrophic activities and created a more reduced condition, which is suitable for denitrifying bacteria and stimulates denitrification. In our study, however, we could observe neither the rise in inorganic N contents as a result of activated nitrification (Table 2) nor microbial respiration as a result of stimulated O_2_ consumption (Fig. 1c) in MS soils.

Decrease in N_2_O emissions by P addition in MS soils could have been due to the stimulated microbial N immobilization and collateral inorganic N consumption, which reduced the resources for producing N_2_O. Hall and Matson (1999, 2003) demonstrated that N addition in a P-limited forest resulted in a 10–100 times greater amount of N_2_O than that from an N-limited forest did. They suggested that P shortage restricted microbial N immobilization and that the surplus N was used by nitrifying and/or denitrifying bacteria, boosting N_2_O emission. According to their hypothesis, P addition will reduce N_2_O emissions because P addition will alleviate the limitation of microbial N immobilization process and subsequently reduce both inorganic N pool and the activities of nitrification and/or denitrification. Sundareshwar et al. (2003) experimentally demonstrated that P addition reduced N_2_O emissions from coastal marsh soils in California via increasing microbial N immobilization and reducing denitrifying activity. In fact our data showed that P addition tended to increase MBN in MS soils (P = 0.07). However, we assume that the decrease in N_2_O emissions by P addition was not caused by the increase in MBN, because the same amount of N resources were available for nitrification and/or denitrification in the P added soils and the control without P addition [see that no significant reductions in NH_4_^+^ and NO_3_^−^ contents were observed in P added soils (Table 2)], although substantial amount of increase in MBN may reduce N_2_O emissions in a longer period.

Instead, we attributed the decrease in N_2_O emissions by P addition to the improvement of respiratory efficiency (both nitrifying and denitrifying respiration). It is well known that nutrient shortage drives microbes to require more energy to maintain their activities and causes a lower efficiency of respiration (Schimel 2003; López-Urrutia and Morán 2007; Sinsabaugh et al. 2013); nutrient supply could increase respiratory efficiency conversely. Since N_2_O is a by-product and an intermediate of nitrifying and denitrifying respiration, respectively (Davidson and Verchot 2000), P addition may increase microbial biomass per N_2_O emission as well as that per CO_2_ emission through improving the respiratory efficiency (López-Urrutia and Morán 2007). In fact, both MBC/CO_2_ ratio and MBC/N_2_O ratio significantly increased by P addition (Table 3) in MS soil, suggesting that the increase in nitrifying and/or denitrifying respiratory efficiency may be a reason for the suppressed N_2_O emissions by P addition in MS soils. We assumed that P addition mainly improved the efficiency of denitrifying respiration, because water condition was adjusted to a relatively high value of WHC 80 % in the present study, where the contribution of nitrification to N_2_O emissions must have been lower compared with that of denitrification (Davidson and Verchot 2000).

As we hypothesized, effects of P addition on N_2_O emissions differed in UB soils from in MS soils. In UB soils, we could not observe any differences in N_2_O emissions between control and P-added soils. Neither MBC/CO_2_ nor MBC/N_2_O changed significantly by P addition. We could not explain about this phenomena from our data. But one assumption is as follows. In UB soils, where the ecosystem processes were more-severely limited by P availability than in MS soils (Kitayama and Aiba 2002), P addition might have changed the microbial community (Li et al. 2010; Liu et al. 2012) from a “high P use efficiency but low growth rate (highly adapted to low P condition) community” to a “lower P use efficiency but higher growth rate (less adapted to low P condition) community” (here we need to admit we did not analyze the microbial community indicators). A higher respiration rate and a higher turnover of the “less adapted to low P condition community” might have resulted in a lowered respiratory efficiency, which offset the promoting effects of P addition on respiratory efficiency (the mechanisms observed in MS soils). Although the assumption is highly speculative, the fact that the initial respiratory efficiency was higher in more-severely P-limited condition in UB soils (Fig. 2b), and the more-severely P-limited condition caused fewer increase in respiratory efficiency (even negative) (Fig. 2c) may support this idea. Based on this idea, Fig. 1c also suggests that the shift in microbial community (*highly adapted to low P condition community* to *less adapted to low P conditioncommunity*) have also occurred in MS soils, because the decrease in ΔMBC/CO_2_ with decreasing P availability was also observed in MS soils. The increase in the respiratory efficiency may have been also partly offset by the shift in soil microbial community in MS soils. According to this idea, the magnitude of offset was probably smaller in MS soils than that in UB soils, which may have caused a clear decrease in N_2_O emissions in P added soils in MS soils. This idea is not based on the data, and needs to be tested in the future. However, at least, we demonstrated that the effects of P addition (or P shortage) on N_2_O emissions may be different depending on the degree of P shortage. Our suggestion may partly explain the inconsistency about the effects of P addition on N_2_O emissions among previous studies (Hall and Matson 1999; Sundareshwar et al. 2003, Mori et al. 2010a, b, 2013c).

Our study also provided a new hypothesis about P shortage in tropical soils (Vitousek and Sanford 1986; Elser et al. 2007) and N_2_O emissions; P shortage in tropical soils (but with ample N) causes a lower nitrifying and/or denitrifying respiratory efficiency, which in turn causes higher N_2_O losses through respiration processes. More data are needed from various types of soils from broader areas to verify or falsify our hypothesis. Changes in microbial community composition by P addition should also be clarified.

## Conclusion

We suggested that P application to the P-limited tropical forest soils enhanced the respiratory efficiency and reduced the gases emitted from respiration (both CO_2_ and N_2_O). We also suggested that the effects of P addition on N_2_O emissions may be different depending on the degree of P shortage. This is the first study that tried to elucidate the factors causing contradictory effects of P addition on N_2_O emission in laboratory condition (without vegetation interaction). Further observations with microbial community analysis using more variety of soils are necessary to fully understand the effects of P addition on N_2_O emissions.
